# Effective BMP-2 Release and Mineralization on a Graphene Oxide/Polyvinylpyrrolidone Hydrogel Forming Poly (ε-Caprolactone) Nanofibrous Scaffolds

**DOI:** 10.3390/ma15238642

**Published:** 2022-12-04

**Authors:** Jin-Oh Jeong, Sung-In Jeong, Youn-Mook Lim, Jong-Seok Park

**Affiliations:** 1 Wake Forest Institute for Regenerative Medicine (WFIRM), Wake Forest School of Medicine, Winston-Salem, NC 27157, USA; 2 Advanced Radiation Technology Institute, Korea Atomic Energy Research Institute (KAERI), Jeongeup-si 56212, Republic of Korea

**Keywords:** PCL nanofibrous scaffolds, drug delivery, gamma-ray irradiation, hydrogel, graphene oxide

## Abstract

PCL nanofibrous scaffolds are widely used as bone scaffolds, and they can increase the efficiency of bone regeneration by loading drugs and/or growth factors onto them. However, to obtain a more effective bone regeneration effect, it is necessary to increase drug loading and release efficiency. In this study, conductive hydrogel forming nanofibrous scaffolds were prepared to increase drug efficiency. GO has an excellent conductivity and biocompatibility, making it an efficient conductive polymer for bone differentiation. Electrospun PCL was immersed in a mixed solution of GO and PVP and then crosslinked using gamma-ray irradiation. It was confirmed that GO/PVP-PCL was successfully prepared through its characterization (morphology, thermal, chemical, electrical, and biological properties). In addition, drug-release efficiency was confirmed by electrical stimulation after loading the sample with BMP-2, a bone-regeneration growth factor. Compared to PCL, it was confirmed that GO/PVP-PCL has an approximately 20% improved drug-release efficiency and an excellent mineralization of the scaffolds using SBF. After culturing MG63 cells on GO/PVP-PCL, a high effect on osteodifferentiation was confirmed by ALP activity. Therefore, GO/PVP-PCL prepared by a gamma-ray-induced crosslinking reaction is expected to be used as biomaterial for bone-tissue engineering.

## 1. Introduction

Electrospun fibrous scaffolds have a very large surface area per unit mass and a morphologically similar structure to the extracellular matrix (ECM), and they are flexible and widely used in tissue engineering [1,2,3,4]. In addition, nanofibrous scaffolds have excellent mechanical properties and are effective bone scaffolds for cell differentiation and proliferation due to their porous structure [5,6,7]. In particular, nanofibrous scaffolds have been studied for their effective tissue regeneration due to the three-dimensional porous structure of the scaffolds for bone-tissue engineering [8,9,10]. The goal of bone-tissue engineering is to develop bone-regenerative materials or to increase the regenerative mechanisms of the human body that can promote bone formation and regeneration [11,12,13]. There are many treatments for bone regeneration through autografts or allografts for bone damage [14,15]. In addition, ceramic (e.g., hydroxyapatite (HA) and tricalcium phosphate (TCP)) or various nanofiber scaffolds are being developed for bone-tissue engineering [16,17]. However, for effective bone regeneration, it is necessary for nanofibrous scaffolds to effectively load and release drugs (e.g., bone morphogenetic protein (BMP), cholecalciferol, ascorbate, and β-glycerophosphate) for bone regeneration [18,19]. Accordingly, drugs can be loaded by conjugating a functional group to nanofibrous scaffolds through surface modification [20]. In addition, drugs can be loaded to nanofibrous scaffolds blended with a polymer and/or biomolecules with a functional group, such as collagen, heparin and biopolymers [21,22]. However, not all drugs are completely loaded to functional groups, and many drugs are not even accurately loaded, which is their disadvantage. Therefore, to maximize the drug loading and release effect, hydrogel forming nanofibers and/or electrospun hydrogels have been studied [23,24,25].

A hydrogel is a material in which a water-soluble polymer forms a three-dimensional structure by physical (e.g., hydrogen bonding, Van der Waals force, hydrophobic interaction, and polymer bonding) or chemical (e.g., covalent bonding) bonding [26,27,28]. Hydrogels are widely used as biomaterials in tissue engineering because they have a high water content and a structure similar to that of the ECM [29,30,31,32]. Especially, a drug delivery system using hydrogels has the advantage of a possible effective drug release due to their high moisture content. Previously, we fabricated a polypyrrole (PPy)/polyvinylpyrrolidone (PVP) conductive hydrogel using gamma-ray irradiation to increase the drug-releasing effect of the hydrogel [33]. After preparing the conductive hydrogels by inducing the polymerization reaction of pyrrole and the crosslinking reaction of PVP, simultaneously, we confirmed that the electrical conductivity of PPy and the mechanical and biological properties of the hydrogel were maintained [33]. In addition, we confirmed the release effect of L-ascorbic acid according to electrical conductivity after preparing a silicone/poly (acrylic acid, PAAc) hydrogel using electron-beam irradiation. We confirmed the change in drug release according to electrical stimulation using the ionic conductivity characteristics of PAAc. The cumulative release of L-ascorbic acid increased as the electrical stimulation was increased from 0 to 7 V [34].

In this study, we prepared PCL nanofibrous scaffolds using electrospinning, and then immersed them in a GO/PVP solution to form GO/PVP hydrogels using a gamma-ray-induced crosslinking reaction. They have the characteristics of the nanofibers and the hydrogel. In particular, graphene oxide (GO) has been shown to be a promising material for biological applications because it has good characteristics, including excellent water solubility, amphiphilicity, easy surface functionalization, surface-enhanced Raman scattering (SERS), and fluorescence-quenching ability [35,36,37,38]. In addition, recently, GO has been widely used as a biomaterial for bone-tissue engineering because it promotes the differentiation and proliferation of osteocytes and induces new bone formation [39,40,41]. However, understandings of the exact mechanism of the induction of bone regeneration is still lacking, and studies to elucidate it have been continuously conducted [42]. We characterized GO/PVP-PCL scaffolds, including the morphology, water contact angle, thermal properties, chemical properties, and electrical properties, and then confirmed the drug-release effect according to electrical conductivity by loading BMP-2 to evaluate cell characteristics. In addition, the mineralization of each scaffold was confirmed using stimulated body fluid (SBF), and the effect of GO/PVP on bone regeneration in vitro was confirmed through the alkaline phosphatase (ALP) activity test. Therefore, GO/PVP-PCL hydrogel prepared using the gamma-ray crosslinking reaction could have promise as a biomaterial for bone-tissue engineering.

## 2. Materials and Methods

### 2.1. Materials

Polycaprolactone (PCL, Mn 70,000 to 90,000 g/mol), Polyvinylpyrrolidone (PVP, (C_6_H_9_NO)_n_, molecular weight: 360,000 g/mol), Glycerin (C_3_H_8_O_3_), and graphene oxide (GO, 4 mg/mL, dispersion in H_2_O) were purchased from Sigma-Aldrich (St. Louis, MO, USA). k-Carrageenan (k-C, (C_24_H_36_O_25_S_2_^2−^), Mn = 788.7 g/mol) and Locust Bean Gum (LBG) were obtained from Korea Karagen Co., Ltd. Tetrahydrofuran (THF) and *N*,*N*-dimethyl formamide (DMF) was purchased from Duksan reagent (Ansan, Korea) and Showa Chemical Co. (Tokyo, Japan). Dulbecco’s modified Eagle’s medium (DMEM), Dulbecco’s phosphate-buffered saline (DPBS), and penicillin streptomycin (PS) were obtained from Gibco (Grand Island, NY, USA). Fetal bovine serum (FBS) was purchased from Hyclone (Logan, UT, USA). All other reagents and solvents were of analytical grade and used as received.

### 2.2. Preparation of the GO/PVP Hydrogel Forming the PCL Nanofibrous Scaffolds

A 13 wt% PCL solution was prepared and then dissolved in a mixture of THF and DMF (70:30, *v*/*v*) at room temperature (RT). The prepared PCL solution was loaded into a 12 mL plastic syringe with a blunt stainless-steel needle (20 G, NanoNC, Seoul, Republic of Korea) to place it into an infusion pump (SHB366, Sckjmotor, Shenzhen, China) with a connecting needle to a high-voltage power supply (ESR-200RL, NanoNC, Seoul, Republic of Korea). The electrospinning parameters were as follows: a flow rate of 2 mL/h; an applied voltage of 11.3 kV; and a distance of 15 cm. After fabricating the PCL nanofibrous scaffolds, they were dried at 40 °C for 4 h in a vacuum oven. 

**Figure 1:**
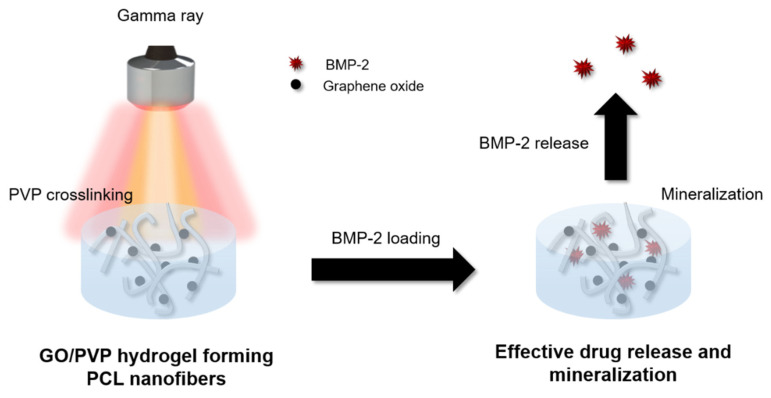
Schematic illustration of the preparation of the GO/PVP hydrogel forming PCL nanofibers.

A 12 wt% PVP solution was prepared and then dissolved in deionized (DI) water using a homogenizer to mix it with 0.5% k-Carrageenan (k-C), 0.25% Locust Bean Gum (LBG), and 1.5% glycerin, and denoted as PVP. Then, the prepared PVP solution was mixed with 10% GO. The PCL nanofibrous scaffolds were immersed in GO containing the PVP (GO/PVP) solution. Then, they were irradiated with a ^60^Co source (ACEL type C-1882, Korea Atomic Energy Research Institute, Jeongeup, Korea) at a dose of 25 kGy (10 kGy/h) as shown in Figure 1. The final product was named GO/PVP-PCL. 

### 2.3. Characterization of the GO/PVP Hydrogel Forming PCL Nanofibrous Scaffolds

The morphology of the PCL and GO/PVP-PCL scaffolds was observed by scanning electron microscopy (SEM, JSM-6390, JEOL, Tokyo, Japan) with an electron beam of 15 to 20 kV and magnification of 1 k. High-resolution images were obtained by sputter-coating with gold for 70 s. 

The wettability of PCL and GO/PVP-PCL was confirmed with a water-contact-angle analyzer (WCA, Surface Electro Optics Co., Suwon, Republic of Korea) according to the static sessile-drop method, applying a drop of DI water on the scaffolds at three or more positions. 

The thermal properties of PCL and GO/PVP-PCL were performed by thermogravimetric analysis (TGA, TA Q600, TA Instrument, Newcastle, PA, USA) with a scaffold weight of 15 mg by placing it in a platinum pan. The heating rate and analyzed temperature were 10 °C/min and 40 to 700 °C under a nitrogen flow.

Differential scanning calorimetry (DSC, TA Q100, TA Instrument, Newcastle, PA, USA) was used to analyze PCL and GO/PVP-PCL with a scaffold weight of 4–6 mg in an aluminum pan at temperatures from 37 to 200 °C (10 °C/min) under a nitrogen atmosphere. 

The chemical properties of PCL and GO/PVP-PCL were measured using ATR-FTIR (attenuated total reflection–Fourier-transformed infrared spectroscopy, Bruker TEMSOR 37, Bruker AXS. Inc., Karlsruhe, Germany) with a range of 500 to 4000 cm^−1^ at a resolution of 4 cm^−1^ averaged over 128 scans. 

The electrical conductivity of PVP and GO/PVP-PCL was measured using a four-point probe method (Modusystems, Hanam, Korea) with the prepared scaffolds (size of 1 cm^2^). Linear scan voltammetry was applied from −1 to 1 V. The thickness was obtained using a Vernier caliper (Mitutoyo, Takatsu-ku, Japan) to determine the resistance from the I-V curve. The electrical conductivity was calculated with the following equation:

Conductivity (S/cm) = 1/(Thickness/Resistance).

### 2.4. BMP-2 Release Test

Bone morphogenetic protein-2 (BMP-2, 25 mg/mL, pepro Tech, Rocky Hill, NJ, USA) was loaded onto PCL and GO/PVP-PCL (1 cm^2^ dimension) after sterilizing them with 70% ethanol for 30 min under UV, and then washed 5 times with PBS. The scaffolds were incubated with the loaded BMP-2 at 4 °C overnight. Then, to perform the BMP-2 release, 10 mL of PBS was put into glass vials, and the solution was stirred with a magnetic stirrer until it reached 37 °C. PCL and GO/PVP-PCL were fixed with an electrode for the electrical stimuli. The cumulative BMP-2 release was induced with a DC power supply (E3634A, Agilent Technologies, Santa Rosa, CA, USA) at 0 and 3 V. Solution samples at time intervals of 0, 10, 20, 30, 40, 50, 60, 120, 240, and 360 min were taken and placed in a 96-well plate. The released BMP-2 solution was measured with an enzyme-linked immunosorbent assay (ELISA) development kit (Pepro Tech, Rocky Hill, NJ, USA) and recorded with a microplate reader at an absorbance of 405 nm.

### 2.5. In Vitro Cytocompatibility

The in vitro cytocompatibility test was performed by the Live/Dead and CCK-8 assays using NIH3T3 cells. NIH3T3 cells (seeding density of 2 × 10^4^ cell/well) were cultured on a 24-trans-well plate in DMEM containing 10% FBS and 1% PS with 5% CO_2_ at 37 °C for 24 h, and then they were loaded onto the scaffolds and incubated for 24 h. After culturing the NIH3T3 cells with the scaffolds for 1 day, live/dead staining (LIVE/DEAD Viability/Cytotoxicity Kit, Molecular Probes Inc., Eugene, OR, USA) was performed by washing the plates with DPBS and then they were stained with calcein-AM and EthD-1 (diluted to 2 and 4 μL with DPBS) and incubated at 37 °C for 15 min. The Live/Dead images were acquired with a fluorescence microscope (DMI3000B, Leica, Wetzlar, Germany). In addition, cell viability was evaluated with the CCK-8 assay. The CCK-8 solution (CCK-8:DMEM = 1:9) was added and then incubated at 5% CO_2_ and 37 °C for 3 h, and the absorbance was measured at 450 nm with a microplate reader (Powerwave XS, Biotek, VT, USA). 

### 2.6. Mineralization and Alkaline phosphatase (ALP) Activity Test

To observe the mineralization on PCL and GO/PVP-PCL, stimulated body fluid (SBF, 1X) was used. The SBF was prepared by mixing NaCl, KCl, CaCl_2_-2H_2_O, MgCl_2_-6H_2_O, NaH_2_PO_4_, Na_2_SO_4_, and NaHCO_3_ in DI water, sequentially. The ion content of blood plasma and the SBF is presented in Table 1. Each scaffold was prepared to a dimension of 2 cm^2^ and immersed in SBF, followed by incubation at 37 °C for 24 h for mineralization. After mineralization, each scaffold was washed with DI water and dried for 24 h. The mineralization of PCL and GO/PVP-PCL was confirmed with SEM images. In addition, ALP activity was evaluated to determine whether PCL and GO/PVP-PCL to which cells were attached affect the role of bone formation by bone differentiation. MG63 cells were seeded onto each scaffold with a seeding density of 1 × 10^5^, and then they were incubated in an osteodifferentiation medium (2.84 × 10^−4^ M L-ascorbic acid, 1 × 10^−2^ M β-glycerol-phosphate) at 37 °C and 5% CO_2_ for 5 days. Then, after an RIPA Lysis buffer (pH 7.2) solution was added and incubated at 4 °C for 20 min, cell-cultured PCL and GO/PVP-PCL were pulverized with a homogenizer. The pulverized scaffolds were mixed with the ALP solution at a ratio of 1:20 and incubated at 37 °C for 30 min, and then the absorbance was measured at 405 nm with a microplate reader (Cytation 5, BioTek, Seoul, Republic of Korea). In addition, the amount of protein in the scaffolds was measured with a Micro BCA kit (Thermo Scientific, Rockford, IL, USA) and a spectrometer at 562 nm.

### 2.7. Statistical Analysis

All tests were performed at least in triplicate, and the data were presented as the mean ± standard deviation (SD) unless otherwise noted. Statistical significance was judged using one-way analysis of variance (ANOVA) with Tukey’s post-hoc comparison of the means with SigmaPlot software (*p* < 0.05, Systat software, Inc., San Jose, CA, USA).

## 3. Results and Discussion

### 3.1. Preparation of the GO/PVP Hydrogel Forming the PCL Nanofibrous Scaffolds

PCL nanofibrous scaffolds were prepared using electrospinning which has good mechanical properties. To increase the efficiency of drug loading and release using the electrical and hydration properties of the hydrogel, GO/PVP was uniformly crosslinked to the PCL scaffolds using gamma-ray irradiation to prepare the hydrogel forming nanofibrous scaffolds. Gamma-ray irradiation can induce various chemical reactions (e.g., polymerization, decomposition, surface modification, and crosslinking) using radical recombination reactions due to their high energy and self-ionization. In addition, it has the advantage of being able to induce reactions in various environments (e.g., oxygen, nitrogen, and vacuum) and states (e.g., solids and liquids) [34,43,44]. Various reactions can be induced by controlling the polymer properties and the conditions of the gamma-ray-irradiation dose and dose rate. Because PVP is a water-soluble polymer, it can be dissolved in water to induce a crosslinking reaction. Radicals are formed on H· and OH· by gamma-ray irradiation, which attacks the C-H group of PVP to form free radicals. The radicals that form on the C-H groups of PVP after gamma-ray irradiation combine to cause crosslinking reactions. Previously, we conducted a study to prepare a hydrogel by inducing a crosslinking reaction of PVP using gamma-ray irradiation [33,45]. Based on previous studies, we conducted research by optimizing the PVP concentration and gamma-ray irradiation dose.

### 3.2. Characterization of the GO/PVP Hydrogel Forming PCL Nanofibrous Scaffolds

**Figure 2:**
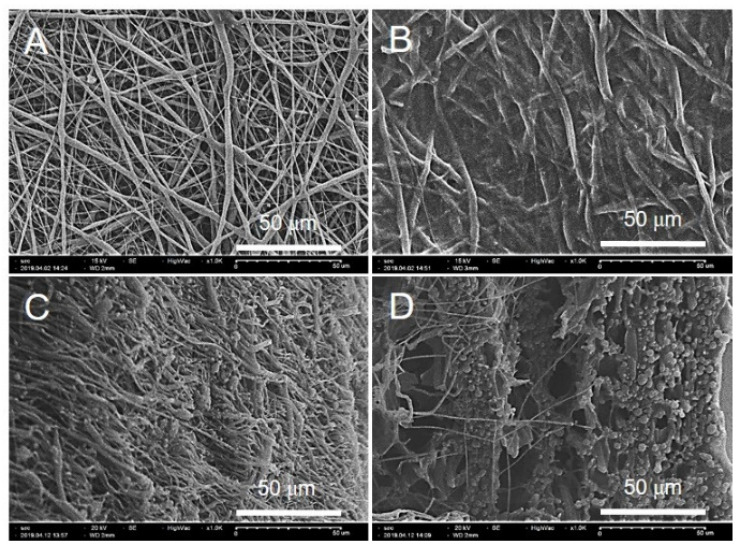
SEM images: (**A**) PCL surface, (**B**) GO/PVP-PCL surface, (**C**) PCL cross-section, and (**D**) GO/PVP-PCL cross-section.

We showed that GO/PVP was uniformly crosslinked into the PCL nanofibers in the SEM image to confirm that it is possible to prepare a hydrogel-type scaffold. As shown in Figure 2A, the morphology of the PCL scaffolds was confirmed as randomly formed fibers, and Figure 2B shows that GO/PVP filled the pores of the fiber surface due to the crosslinking of GO/PVP. In addition, Figure 2D shows that GO/PVP was crosslinked throughout the fibers even in the cross-section of the scaffolds.

**Figure 3:**
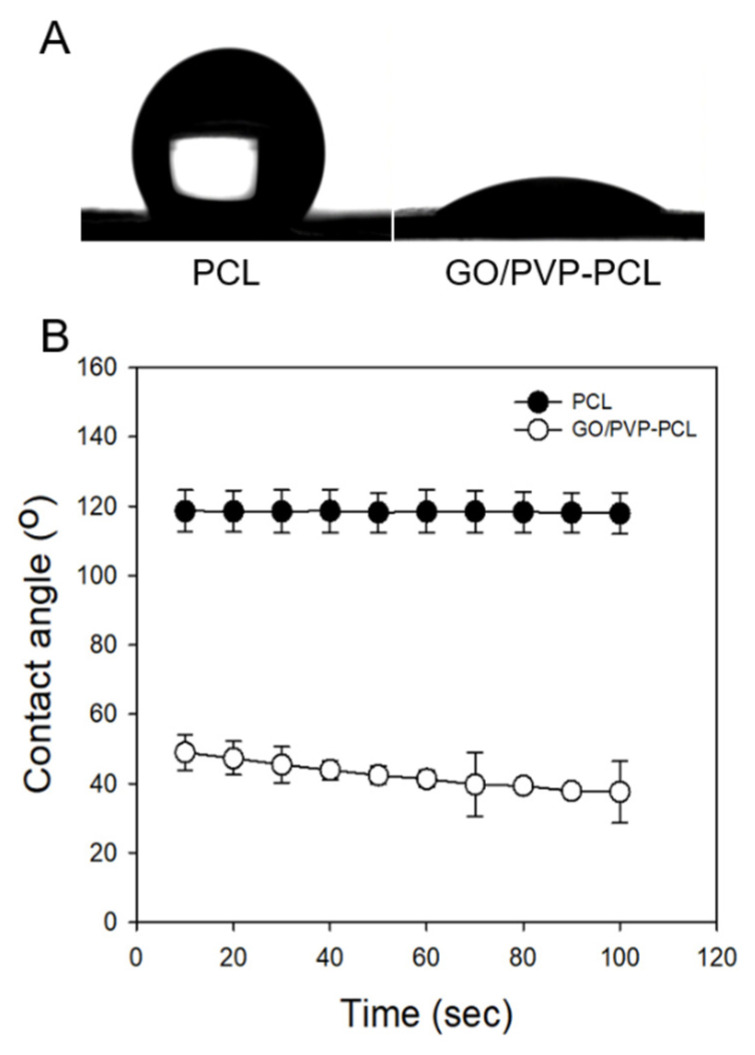
Water contact angle (WCA) of PCL and GO/PVP-PCL: (**A**) WCA images; (**B**) WCA graph.

PCL is characterized by high hydrophobicity. The WCA of PCL was 118.62 ± 6.00°, and it maintained high hydrophobicity at 117.91 ± 5.99°, even after 100 s (Figure 3). On the other hand, GO/PVP-PCL had a low WCA of 48.94 ± 25.11° due to the hydrophilic characteristics of GO and PVP. In addition, after observing the WCA at 100 s, GO/PVP-PCL slightly decreased to 37.59 ± 18.92. This result shows that GO/PVP was successfully crosslinked by gamma-ray irradiation, making it hydrophilic instead of the original hydrophobic properties of the PCL scaffolds. It was confirmed through the WCA that GO/PVP-PCL exhibits hydrophilic properties compared to hydrophobic PCL. 

**Figure 4:**
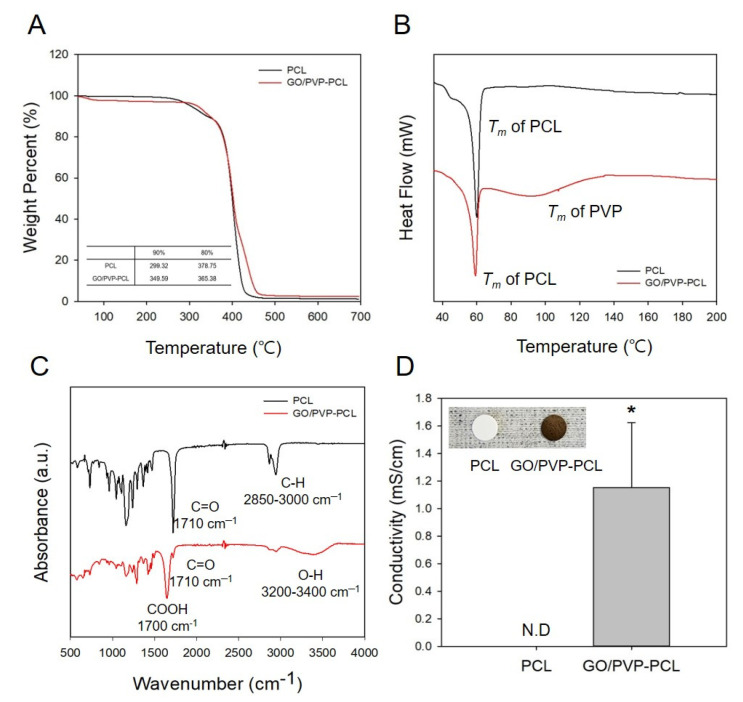
Characterization of PCL and GO/PVP-PCL: (**A**) Thermogravimetric analysis (TGA); (**B**) Differen-tial scanning calorimetry (DSC); (**C**) FTIR spectra; (**D**) Conductivity.

The thermal properties of PCL and GO/PVP-PCL were confirmed by TGA (Figure 4A) and DSC (Figure 4B). PCL showed that it started to degrade at about 300 °C and completely disappeared at 400 °C. On the other hand, the degradation of GO/PVP-PCL started at about 350 °C and completely disappeared at 450 °C. This result suggests that the annihilation of GO/PVP-PCL at a higher temperature than PCL was due to the stable thermal properties of GO. In addition, as shown in Figure 4B, the T_m_ of PCL and GO/PVP-PCL was confirmed with DSC. The peak of PCL was confirmed at 60 °C because the T_m_ of general PCL is 60 °C, and in the case of GO/PVP-PCL, the T_m_ peak of PVP was confirmed between 60 °C and 120 °C. Therefore, the results of the thermal property analysis (i.e., TGA and DSC) showed that GO/PVP was successfully infiltrated and crosslinked to the PCL scaffolds. In addition, ATR-FTIR was conducted to confirm the chemical structures of PCL and GO/PVP-PCL as shown in Figure 4C. The general chemical structural peaks of PCL were C=O and C-H, identified at 1710 cm^−1^ and 2850–3000 cm^−1^, respectively. In addition, the COOH- and O-H groups of GO/PVP-PCL were confirmed at 1700 cm^−1^ and 3200–3400 cm^−1^, respectively. This result shows that the COOH- and O-H groups appearing in the FTIR spectra were due to GO. By evaluating the thermal and chemical properties, it was confirmed that their properties were maintained even when GO/PVP was crosslinked to the PCL scaffolds through gamma-ray-induced crosslinking reactions. In addition, the electrical properties of the scaffolds were confirmed because of GO, and the conductivity could not be confirmed in PCL because there was no electrical property; however, the conductivity of GO/PVP-PCL was 1.15 ± 0.47 mS/cm (Figure 4D). Therefore, the thermal, chemical, and electrical properties showed that GO/PVP was successfully infiltrated and crosslinked to the PCL scaffold. In particular, the electrical properties of GO were maintained even after irradiation with gamma-rays, evidenced by the conductivity of GO/PVP-PCL. Previously, we conducted a study to optimize the conditions by simultaneously inducing the polymerization reaction of polypyrrole and the crosslinking reaction of PVP by gamma-ray irradiation. This study confirmed that pyrrole was polymerized to PPy by gamma-ray irradiation and that its electrical properties were maintained. The π–π conjugation of conducting polymers can have the weakness of being easily broken by high energy, such as gamma-rays. However, we fabricated materials that maintain the electrical properties of PPy by controlling parameters, such as the concentration of conducting polymer, dopant contents, irradiation dose, dose rate, and the irradiation environment [33]. Thus, GO was also confirmed to maintain its conductivity by optimizing the conditions in which the electrical properties were not changed by gamma-ray irradiation and were maintained. 

### 3.3. In Vitro Cytocompatibility

**Figure 5:**
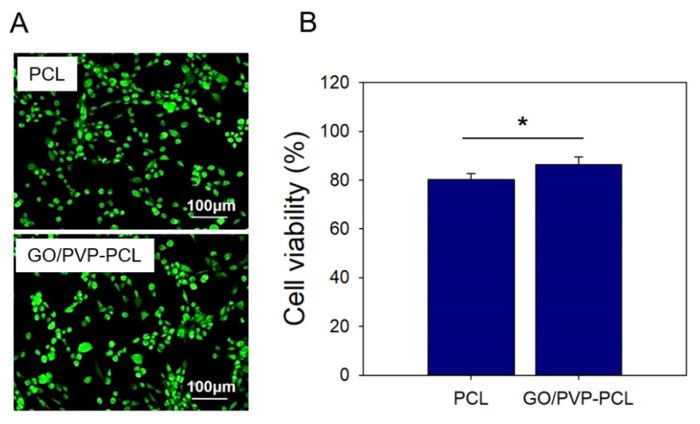
In vitro cytocompatibility of PCL and GO/PVP-PCL: (**A**) Live/Dead assay; (**B**) CCK assay. “*” Indicates statistical significance relative to the PCL (*p* < 0.05).

In vitro cytocompatibility of PCL and GO/PVP-PCL was confirmed with NIH3T3 cells. Figure 5A shows an image of the Live/Dead assay on day 1. In the case of PCL, although it has hydrophobic properties, it was confirmed that a large number of live cells were present due to the PCL characteristics as a biodegradable polymer. In addition, GO/PVP-PCL showed a large number of live cells, similar to the PCL result, because GO and PVP are also biopolymers. In Figure 5B, the cell viability of each sample was confirmed with the CCK-8 assay. Similar to the Live/Dead assay results, PCL and GO/PVP-PCL showed a high cell viability of over 80%. 

### 3.4. BMP-2 Release Test

**Figure 6:**
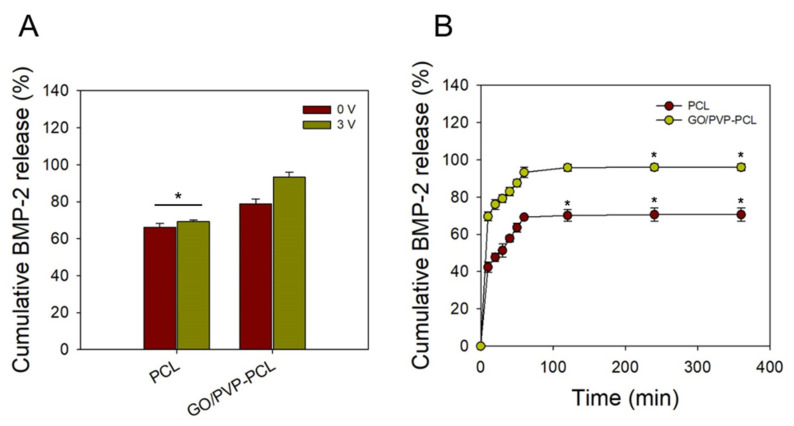
In vitro BMP-2 release from the BMP-2-loaded PCL and GO/PVP-PCL at different voltages: (**A**) Cumulative BMP-2 release at different voltages (0 and 3 V) for 60 min. (**B**) Cumulative BMP-2 release for different time point (10, 20, 30, 40, 50, 60, 120, 240, and 360 min) at 3 V. “*” Indicates statistical significance relative to the 0 V and PCL (*p* < 0.05).

BMP-2 is a potential osteoclast derivative that strongly induces autologous and heterologous bone formation in vivo and differentiates precursors and/or undifferentiated stem cells into osteoblasts in vitro [46,47] In particular, as the clinical use of BMP-2 has increased, side effects, such as inflammation and ectopic bone formation, are increasing. However, clinical trials are essential to minimize side effects [48]. In this study, a scaffold capable of promoting bone differentiation by increasing the drug delivery efficiency of BMP-2 was prepared. According to the electrical properties of GO, the drug-release behavior of BMP-2 was confirmed with different voltages. Figure 6A shows that the cumulative drug release was confirmed according to the different voltages (i.e., 0 and 3 V) applied to PCL and GO/PVP-PCL at 60 min. In the case of PCL, the drug-release amount at 0 and 3 V was 66.12 ± 2.24% and 69.25 ± 0.79%, respectively. Because PCL has no electrical properties, there was no change in the drug release according to the different voltages. On the other hand, in the case of GO/PVP-PCL, the drug-release amount was 78.73 ± 2.78% at 0 V, and a high drug-release rate of 93.28 ± 2.70% was confirmed at 3 V. Because GO/PVP-PCL is a hydrogel forming a nanofibrous scaffold, it is thought to be effective for drug loading and release. When electrical stimulation was applied, GO was effective in releasing the drug. Figure 6B shows a graph confirming the drug-release behavior over time when electrical stimulation was applied to all samples. As seen in the results of the cumulative BMP-2 release over 360 min, the drug-release amount increased until 60 min and then became saturated from 60 to 360 min. The drug release was increased by electrical stimulation due to the electrical properties of GO. Even without electrical stimulation, GO/PVP-PCL showed a higher drug release than that of PCL because of the hydrogel forming scaffolds. The drugs are charged by a microcurrent and can be easily released because the drug molecules charged by the electric force are rapidly released according to the electric field. Therefore, it is possible to control drug-release efficiency according to the conductivity. [45].

### 3.5. Mineralization and ALP Activity Test

**Figure 7:**
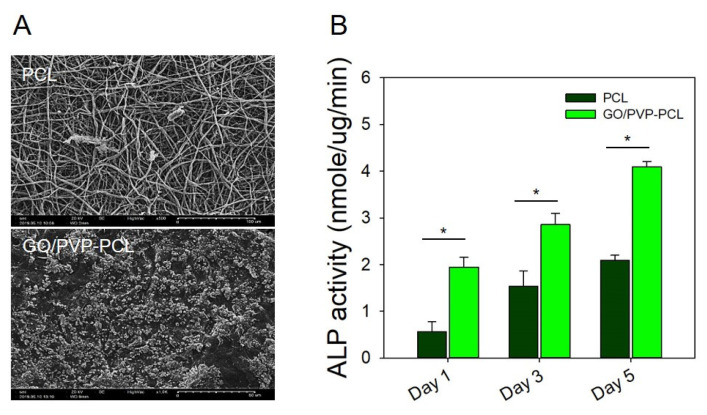
(**A**) SEM images (magnification of ×500 (100 μm) and ×1000 (50 μm)) of the mineralization using 1X SBF solution, (**B**) ALP activity of PCL and GO/PVP-PCL. “*” Indicates statistical significance relative to the PCL (*p* < 0.05).

Especially, GO has been used for stem cell cultures and osteogenic differentiation studies because of its biocompatibility and electrical conductivity properties [49,50]. Recently, it has been reported that the neuronal differentiation of human mesenchymal stem cells (hMSC) was enhanced using GO because it can be applied to the cell adhesion layer, which has an electrical-signal-transduction effect according to the electrical stimulation of GO during cell differentiation [51]. In addition, GO is known to further enhance osteogenic differentiation compared to general growth factors. We confirmed mineralization by loading all the samples into SBF, and a large amount of mineralization was observed by GO/PVP. In addition, after culturing MG63 (osteodifferentiated cells) on the scaffolds, we confirmed the osteodifferentiation effects by ALP activity. The bone differentiation characteristics of GO and mineralization according to the hydrogel form of PVP were confirmed with SBF by SEM analysis. Figure 7A shows an image of PCL in which mineralization hardly occurred. On the other hand, in the case of GO/PVP-PCL, it was confirmed that a lot of mineralization occurred filling the scaffold. This result suggests that effective mineralization proceeded according to the osteogenic properties of GO and the hydrogel form of PVP. In addition, Figure 7B shows whether the scaffolds to which MG63 cells were attached affected the role of bone formation by bone differentiation. The ALP activity of PCL at 1, 3, and 5 days was 0.56 ± 0.21, 1.54 ± 0.32, and 2.08 ± 0.11 nmole/ug/min, respectively, confirming that ALP activity increased. In addition, the ALP activity of GO/PVP-PCL at 1, 3, and 5 days was 1.94 ± 0.21, 2.86 ± 0.24, and 4.09 ± 0.11 nmole/ug/min, respectively, confirming a higher bone differentiation than that of PCL. These results are considered to be indicative of high bone differentiation because the differentiation and proliferation of the MG63 cells attached to the scaffolds were increased by the hydrogel form of GO and PVP. According to the characteristics of GO to improve bone differentiation and the hydrogel forming nanofibrous scaffolds, a higher effect on bone differentiation was confirmed than that of PCL over time from 1 to 5 days.

## 4. Conclusions

We developed conductive hydrogel forming nanofibrous scaffolds for effective drug delivery and good mineralization in bone-tissue engineering using gamma-ray-induced crosslinking reactions. Electrospun PCL was prepared by electrospinning, and then GO/PVP-PCL was successfully prepared using gamma-ray irradiation with its hydrogel forming nanofibrous scaffolds. GO/PVP-PCL was shown to have a higher BMP-2 drug release than that of PCL by electrical stimulation. Because GO/PVP-PCL is in a hydrogel state, the drug loading and release were efficient. In addition, the drug-release efficiency of GO/PVP-PCL increased by approximately 20% compared to PCL by electrical stimulation (voltage at 3 V) because of the electrical properties of GO. Furthermore, efficient mineralization was confirmed through SEM, and the excellent effect on osteodifferentiation was confirmed through ALP activity due to the efficient osteogenic properties of GO. Therefore, it is possible to prepare conductive hydrogel forming nanofibrous scaffolds, and GO/PVP-PCL scaffolds could be promising scaffolds for bone-tissue engineering applied to various specific bone targets.

## Figures and Tables

**Table 1:** Ion-content in human blood plasma and SBF.

Ion		Na^+^	K^+^	Mg^2+^	Ca^2+^	Cl^−^	HCO_3_^−^	HPO_4_^2−^	SO_4_^2−^	pH
Ion-content (mM)	Blood plasma	142.0	5.0	1.5	2.5	103.0	27.0	1.0	0.5	7.2~7.4
	SBF	142.0	5.0	1.5	2.5	147..8	4.2	1.0	0.5	7.4

## Data Availability

The data presented in this study are available on request from the corresponding author.

## References

[1] Liao S., Li B., Ma Z., Wei H., Chan C., Ramakrishna S. (2006). Biomimetic Electrospun Nanofibers for Tissue Regeneration. Biomed. Mater..

[2] Barnes C.P., Sell S.A., Boland E.D., Simpson D.G., Bowlin G.L. (2007). Nanofiber Technology: Designing the next Generation of Tissue Engineering Scaffolds. Adv. Drug Deliv. Rev..

[3] Venugopal J., Low S., Choon A.T., Ramakrishna S. (2008). Interaction of Cells and Nanofiber Scaffolds in Tissue Engineering. J. Biomed. Mater. Res. Part B Appl. Biomater..

[4] Smith L.A., Ma P.X. (2004). Nano-Fibrous Scaffolds for Tissue Engineering. Colloids Surf. B Biointerfaces.

[5] Xu T., Miszuk J.M., Zhao Y., Sun H., Fong H. (2015). Electrospun Polycaprolactone 3D Nanofibrous Scaffold with Interconnected and Hierarchically Structured Pores for Bone Tissue Engineering. Adv. Healthc. Mater..

[6] Khajavi R., Abbasipour M., Bahador A. (2016). Electrospun Biodegradable Nanofibers Scaffolds for Bone Tissue Engineering. J. Appl. Polym. Sci..

[7] Gao Y., Shao W., Qian W., He J., Zhou Y., Qi K., Wang L., Cui S., Wang R. (2018). Biomineralized Poly (l-Lactic-Co-Glycolic Acid)-Tussah Silk Fibroin Nanofiber Fabric with Hierarchical Architecture as a Scaffold for Bone Tissue Engineering. Mater. Sci. Eng. C.

[8] Lin W., Chen M., Qu T., Li J., Man Y. (2020). Three-Dimensional Electrospun Nanofibrous Scaffolds for Bone Tissue Engineering. J. Biomed. Mater. Res. Part B Appl. Biomater..

[9] Yao Q., Cosme J.G.L., Xu T., Miszuk J.M., Picciani P.H.S., Fong H., Sun H. (2017). Three Dimensional Electrospun PCL/PLA Blend Nanofibrous Scaffolds with Significantly Improved Stem Cells Osteogenic Differentiation and Cranial Bone Formation. Biomaterials.

[10] Ye K., Liu D., Kuang H., Cai J., Chen W., Sun B., Xia L., Fang B., Morsi Y., Mo X. (2019). Three-Dimensional Electrospun Nanofibrous Scaffolds Displaying Bone Morphogenetic Protein-2-Derived Peptides for the Promotion of Osteogenic Differentiation of Stem Cells and Bone Regeneration. J. Colloid Interface Sci..

[11] Pilipchuk S.P., Plonka A.B., Monje A., Taut A.D., Lanis A., Kang B., Giannobile W.V. (2015). Tissue Engineering for Bone Regeneration and Osseointegration in the Oral Cavity. Dent. Mater..

[12] Koons G.L., Diba M., Mikos A.G. (2020). Materials Design for Bone-Tissue Engineering. Nat. Rev. Mater..

[13] Li J.J., Ebied M., Xu J., Zreiqat H. (2018). Current Approaches to Bone Tissue Engineering: The Interface between Biology and Engineering. Adv. Healthc. Mater..

[14] Nazirkar G., Singh S., Dole V., Nikam A. (2014). Effortless Effort in Bone Regeneration: A Review. J. Int. Oral Health.

[15] Matassi F., Nistri L., Chicon Paez D., Innocenti M. (2011). New Biomaterials for Bone Regeneration. Clin. Cases Min. Bone Metab..

[16] Wen Y., Xun S., Haoye M., Baichuan S., Peng C., Xuejian L., Kaihong Z., Xuan Y., Jiang P., Shibi L. (2017). 3D Printed Porous Ceramic Scaffolds for Bone Tissue Engineering: A Review. Biomater. Sci..

[17] Li X., Wang L., Fan Y., Feng Q., Cui F.-Z., Watari F. (2013). Nanostructured Scaffolds for Bone Tissue Engineering. J. Biomed. Mater. Res. Part A.

[18] Ghosh S., Webster T.J. (2021). Mesoporous Silica Based Nanostructures for Bone Tissue Regeneration. Front. Mater..

[19] Amini A.R., Laurencin C.T., Nukavarapu S.P. (2012). Bone Tissue Engineering: Recent Advances and Challenges. Crit. Rev. Biomed. Eng..

[20] Sill T.J., von Recum H.A. (2008). Electrospinning: Applications in Drug Delivery and Tissue Engineering. Biomaterials.

[21] Zhang Y., Su B., Venugopal J., Ramakrishna S., Lim C. (2007). Biomimetic and Bioactive Nanofibrous Scaffolds from Electrospun Composite Nanofibers. Int. J. Nanomed..

[22] Zhang Q., Li Y., Lin Z.Y.W., Wong K.K.Y., Lin M., Yildirimer L., Zhao X. (2017). Electrospun Polymeric Micro/Nanofibrous Scaffolds for Long-Term Drug Release and Their Biomedical Applications. Drug Discov. Today.

[23] Bayer I.S. (2021). A Review of Sustained Drug Release Studies from Nanofiber Hydrogels. Biomedicines.

[24] Park S.J., Lee Y.J., Heo D.N., Kwon I.K., Yun K.-S., Kang J.Y., Lee S.H. (2015). Functional Nerve Cuff Electrode with Controllable Anti-Inflammatory Drug Loading and Release by Biodegradable Nanofibers and Hydrogel Deposition. Sens. Actuators B Chem..

[25] Pawłowska S., Rinoldi C., Nakielski P., Ziai Y., Urbanek O., Li X., Kowalewski T.A., Ding B., Pierini F. (2020). Ultraviolet Light-Assisted Electrospinning of Core–Shell Fully Cross-Linked P(NIPAAm-Co-NIPMAAm) Hydrogel-Based Nanofibers for Thermally Induced Drug Delivery Self-Regulation. Adv. Mater. Interfaces.

[26] Kopeček J., Yang J. (2007). Hydrogels as Smart Biomaterials. Polym. Int..

[27] Mathur A.M., Moorjani S.K., Scranton A.B. (1996). Methods for Synthesis of Hydrogel Networks: A Review. J. Macromol. Sci. Part C.

[28] Ahmed E.M. (2015). Hydrogel: Preparation, Characterization, and Applications: A Review. J. Adv. Res..

[29] Liu M., Zeng X., Ma C., Yi H., Ali Z., Mou X., Li S., Deng Y., He N. (2017). Injectable Hydrogels for Cartilage and Bone Tissue Engineering. Bone Res..

[30] Drury J.L., Mooney D.J. (2003). Hydrogels for Tissue Engineering: Scaffold Design Variables and Applications. Biomaterials.

[31] Zhu J., Marchant R.E. (2011). Design Properties of Hydrogel Tissue-Engineering Scaffolds. Expert Rev. Med. Devices.

[32] Tan H., Marra K.G. (2010). Injectable, Biodegradable Hydrogels for Tissue Engineering Applications. Materials.

[33] Jeong J.-O., Park J.-S., Kim Y.-A., Yang S.-J., Jeong S.-I., Lee J.-Y., Lim Y.-M. (2020). Gamma Ray-Induced Polymerization and Cross-Linking for Optimization of PPy/PVP Hydrogel as Biomaterial. Polymers.

[34] Kim Y.-A., Jeong J.-O., Park J.-S. (2021). Preparation and Characterization of Ionic Conductive Poly(Acrylic Acid)-Based Silicone Hydrogels for Smart Drug Delivery System. Polymers.

[35] Huang X., Yin Z., Wu S., Qi X., He Q., Zhang Q., Yan Q., Boey F., Zhang H. (2011). Graphene-Based Materials: Synthesis, Characterization, Properties, and Applications. Small.

[36] Georgakilas V., Tiwari J.N., Kemp K.C., Perman J.A., Bourlinos A.B., Kim K.S., Zboril R. (2016). Noncovalent Functionalization of Graphene and Graphene Oxide for Energy Materials, Biosensing, Catalytic, and Biomedical Applications. Chem. Rev..

[37] Khatir N.M., Fatoorehchi H., Ahmadi A., Khoshnoodfar A., Faghihnasiri M. (2021). Investigating the Adsorption of the Thyroid Stimulating Hormones Molecules on Graphene Sheets by the Density Functional Theory for Possible Nano-Biosensor Applications. J. Chem. Pet. Eng..

[38] Khatir N.M., Ahmadi A., Taghizade N., Motevali Khameneh S., Faghihnasiri M. (2020). Electronic Transport Properties of Nanoribbons of Graphene and ψ-Graphene -Based Lactate Nanobiosensor. Superlattices Microstruct..

[39] Dinescu S., Ionita M., Ignat S.-R., Costache M., Hermenean A. (2019). Graphene Oxide Enhances Chitosan-Based 3D Scaffold Properties for Bone Tissue Engineering. Int. J. Mol. Sci..

[40] Shadjou N., Hasanzadeh M. (2016). Graphene and Its Nanostructure Derivatives for Use in Bone Tissue Engineering: Recent Advances. J. Biomed. Mater. Res. Part A.

[41] Nie W., Peng C., Zhou X., Chen L., Wang W., Zhang Y., Ma P.X., He C. (2017). Three-Dimensional Porous Scaffold by Self-Assembly of Reduced Graphene Oxide and Nano-Hydroxyapatite Composites for Bone Tissue Engineering. Carbon.

[42] Holt B.D., Wright Z.M., Arnold A.M., Sydlik S.A. (2017). Graphene Oxide as a Scaffold for Bone Regeneration. WIREs Nanomed. Nanobiotechnol..

[43] Kim S., Jeong J.-O., Lee S., Park J.-S., Gwon H.-J., Jeong S.I., Hardy J.G., Lim Y.-M., Lee J.Y. (2018). Effective Gamma-Ray Sterilization and Characterization of Conductive Polypyrrole Biomaterials. Sci. Rep..

[44] Jeong J.-O., Jeong S.I., Park J.-S., Gwon H.-J., Ahn S.-J., Shin H., Lee J.Y., Lim Y.-M. (2017). Development and Characterization of Heparin-Immobilized Polycaprolactone Nanofibrous Scaffolds for Tissue Engineering Using Gamma-Irradiation. RSC Adv..

[45] Yang S.-J., Jeong J.-O., Lim Y.-M., Park J.-S. (2021). Synthesis and Characterization of PVP Microneedle Patch Using Metal Bioelectrodes for Novel Drug Delivery System. Mater. Des..

[46] Prall W.C., Haasters F., Heggebö J., Polzer H., Schwarz C., Gassner C., Grote S., Anz D., Jäger M., Mutschler W. (2013). Mesenchymal Stem Cells from Osteoporotic Patients Feature Impaired Signal Transduction but Sustained Osteoinduction in Response to BMP-2 Stimulation. Biochem. Biophys. Res. Commun..

[47] Rickard D.J., Sullivan T.A., Shenker B.J., Leboy P.S., Kazhdan I. (1994). Induction of Rapid Osteoblast Differentiation in Rat Bone Marrow Stromal Cell Cultures by Dexamethasone and BMP-2. Dev. Biol..

[48] James A.W., LaChaud G., Shen J., Asatrian G., Nguyen V., Zhang X., Ting K., Soo C. (2016). A Review of the Clinical Side Effects of Bone Morphogenetic Protein-2. Tissue Eng. Part B Rev..

[49] Fu C., Bai H., Zhu J., Niu Z., Wang Y., Li J., Yang X., Bai Y. (2017). Enhanced Cell Proliferation and Osteogenic Differentiation in Electrospun PLGA/Hydroxyapatite Nanofibre Scaffolds Incorporated with Graphene Oxide. PLoS ONE.

[50] Halim A., Luo Q., Ju Y., Song G. (2018). A Mini Review Focused on the Recent Applications of Graphene Oxide in Stem Cell Growth and Differentiation. Nanomaterials.

[51] Rawat S., Jain K.G., Gupta D., Raghav P.K., Chaudhuri R., Pinky, Shakeel A., Arora V., Sharma H., Debnath D. (2021). Graphene Nanofiber Composites for Enhanced Neuronal Differentiation of Human Mesenchymal Stem Cells. Nanomedicine.

